# Lurbinectedin induces depletion of tumor-associated macrophages, an essential component of its *in vivo* synergism with gemcitabine, in pancreatic adenocarcinoma mouse models

**DOI:** 10.1242/dmm.026369

**Published:** 2016-12-01

**Authors:** María Virtudes Céspedes, María José Guillén, Pedro Pablo López-Casas, Francesca Sarno, Alberto Gallardo, Patricia Álamo, Carmen Cuevas, Manuel Hidalgo, Carlos María Galmarini, Paola Allavena, Pablo Avilés, Ramón Mangues

**Affiliations:** 1 Institut d'Investigacions Biomèdiques Sant Pau, CIBER de Bioingenieria, Biomateriales y Nanomedicina (CIBER-BBN) and Josep Carreras Research Institute, Hospital de Sant Pau, Av. Sant Antoni M. Claret, 167, Barcelona 08025, Spain; 2 Department of Research and Development (R&D), PharmaMar S.A, Av. de los Reyes, 1, Colmenar Viejo, Madrid 28770, Spain; 3 Centro Nacional de Investigaciones Oncológicas (CNIO), Calle de Melchor Fernandez Almagro, 3, Madrid 28029, Spain; 4 IRCCS Istituto Clinico Humanitas, via Manzoni 56, Rozzano, Milano 20089, Italy

**Keywords:** PDA mouse models, Lurbinectedin, Gemcitabine, Synergism, Tumor-associated macrophage depletion

## Abstract

We explored whether the combination of lurbinectedin (PM01183) with the antimetabolite gemcitabine could result in a synergistic antitumor effect in pancreatic ductal adenocarcinoma (PDA) mouse models. We also studied the contribution of lurbinectedin to this synergism. This drug presents a dual pharmacological effect that contributes to its *in vivo* antitumor activity: (i) specific binding to DNA minor grooves, inhibiting active transcription and DNA repair; and (ii) specific depletion of tumor-associated macrophages (TAMs). We evaluated the *in vivo* antitumor activity of lurbinectedin and gemcitabine as single agents and in combination in SW-1990 and MIA PaCa-2 cell-line xenografts and in patient-derived PDA models (AVATAR). Lurbinectedin-gemcitabine combination induced a synergistic effect on both MIA PaCa-2 [combination index (CI)=0.66] and SW-1990 (CI=0.80) tumor xenografts. It also induced complete tumor remissions in four out of six patient-derived PDA xenografts. This synergism was associated with enhanced DNA damage (anti-γ-H2AX), cell cycle blockage, caspase-3 activation and apoptosis. In addition to the enhanced DNA damage, which is a consequence of the interaction of the two drugs with the DNA, lurbinectedin induced TAM depletion leading to cytidine deaminase (CDA) downregulation in PDA tumors. This effect could, in turn, induce an increase of gemcitabine-mediated DNA damage that was especially relevant in high-density TAM tumors. These results show that lurbinectedin can be used to develop ‘molecularly targeted’ combination strategies.

## Abstract (precis)

**Summary:** Lurbinectedin-gemcitabine synergism in PDA models involves lurbinectedin-induced tumor macrophage depletion, triggering CDA downregulation and enhanced DNA damage, supporting the use of this combination to treat macrophage-infiltrated pancreatic tumors.

## INTRODUCTION

Lurbinectedin (PM01183) is a new anticancer agent that displays a potent *in vitro* activity against a broad panel of human-derived tumor cell lines, with growth-inhibition 50% (GI50) concentrations within the picomolar range (Leal et al., 2010). The drug also shows significant antitumor activity in lung, ovarian, colorectal and gastric carcinoma xenografts, among others. At the clinical level, lurbinectedin is currently being evaluated in late-stage (Phase II and III) trials for patients with solid tumors such as platinum-resistant ovarian cancer, BRCA-1/2 mutated breast cancer and small-cell lung cancer. Lurbinectedin is a tetrahydroisoquinoline that reacts through its hemiaminal moiety with the exocyclic amino group of specific guanines in the minor groove of DNA, forming a covalent bond (Leal et al., 2010; Bueren-Calabuig et al., 2011). Its sequence specificity depends on the establishment of highly specific hydrogen bonds with the nucleotides both sides of the guanine. Lurbinectedin-DNA adducts then induce a cascade of events leading to the specific and rapid degradation of the largest subunit of Rpb1 via the ubiquitin-proteasome pathway, and to the inhibition of the nucleotide-excision repair (NER) system (Soares et al., 2011). These effects finally give rise to single-stranded or double-stranded DNA (dsDNA) breaks (SSBs and DSBs, respectively). The accumulation of DNA damage delays progression through the S/G2 phase of the cell cycle and, ultimately, triggers caspase-dependent apoptotic death (Leal et al., 2010; Soares et al., 2012). In addition, lurbinectedin antitumor activity was also related to the depletion of tumor-associated macrophages (TAMs) in different tumor models (Romano et al., 2013; Germano et al., 2013).

Nowadays, the improved understanding of the mechanism of action of anticancer agents is used to establish what it can be called ‘intelligent’ combinations. According to this idea, it would be possible to develop ‘molecularly targeted’ strategies to increase the therapeutic index of a drug combination in order to achieve the best synergistic effects, concentrating the pharmacodynamic effects on tumor cells and thus avoiding unnecessary toxicities. Based on the novelty of its mechanism of action, lurbinectedin is excellently suited for this strategy. For instance, the ability of lurbinectedin to inhibit the NER has been the basis for its rational combination with cisplatin, because this last drug is more active in NER-deficient cells. The hypothesis proved to be right: a synergistic effect of the lurbinectedin-cisplatin combination was demonstrated in both cisplatin-resistant (with high NER activity) and parental ovarian carcinoma cell lines, *in vitro* (Soares et al., 2011) and *in vivo* (Vidal et al., 2012). Lurbinectedin has also been combined with gemcitabine, a pyrimidine analog that, after intracellular conversion to its triphosphate form, incorporates into DNA, leading to DNA strand termination and apoptosis induction (Galmarini et al., 2002; Mini et al., 2006). The aim of this study is to explore whether the lurbinectedin-gemcitabine combination was synergistic in cell-line-derived pancreatic ductal adenocarcinoma (PDA) xenograft and in patient-derived PDA (AVATAR) mouse models, and to study the mechanisms that could explain the synergism, including changes in pharmacological markers such as γ-H2AX foci formation (as a DSB surrogate marker), capase-3 activation, apoptosis induction and/or inhibition of proliferation. In addition, the lurbinectedin-induced selective killing of TAMs and its contribution to the synergism was also determined.

## RESULTS

### Lurbinectedin and gemcitabine treatments are synergistic *in vivo* in SW-1990 and MIA PaCa-2 xenografts

**Fig. 1.**
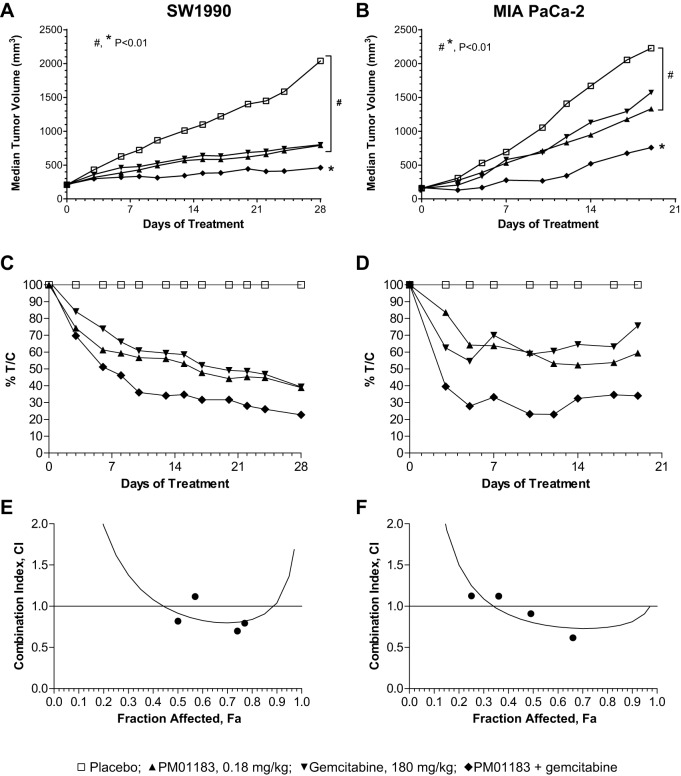
***In vivo* characterization of the synergistic effect of lurbinectedin (PM01183) combined with gemcitabine.** Nude athymic mice bearing subcutaneous tumors (SW-1990 or MIA PaCa-2) sized ca. 150 mm^3^ were randomly allocated and treated with PM01183 (0.180 mg kg^−1^), gemcitabine (180.0 mg kg^−1^) or its combination [PM01183 plus gemcitabine (0.180 mg kg^−1^+180.0 mg kg^−1^)]. (A,B) Tumor growth (median) curves for mice bearing SW-1190 (A) or MIA PaCa-2 (B) tumors and treated with the highest doses of PM01183, gemcitabine or its combination. (C,D) Antitumor activity of each single or combined high-dosed treatment followed by *T*/*C* values, defined as the change in tumor volume for each treated (*T*) and placebo (*C*) group during the placebo-treated survival period for mice bearing SW-1190 (C) or MIA PaCa-2 (D). (E,F) Determination of tumor fraction affected (*F*_a_) by treatments, calculated according to the formula *F*_a_=1– *T*/*C* and combination index (CI) determined by the CI-isobol method for mice bearing SW-1190 (E) or MIA PaCa-2 (F). Statistically significant differences at *P*<0.01 (two-tailed Mann–Whitney U-test).

The *in vivo* antitumor activity of lurbinectedin or gemcitabine as single agents or the synergy of their combination was explored in mice implanted with two different cell lines of human PDA, namely SW-1990 and MIA PaCa-2. Fig. 1A and Fig. S1A show the tumor growth curve for animals bearing SW-1990 tumors at different dose levels. At the highest dose, both lurbinectedin (0.180 mg kg^−1^; all values are given in per kg body weight) and gemcitabine (180.0 mg kg^−1^) displayed similar antitumor activity (Fig. 1A). At the end of the follow-up period (day 28), the median interquartile range (IQR) tumor volume (mm^3^) was 2040 (range 1898 to 2149), 792.6 (range 651.8 to 953.3) and 802.5 (range 668.3 to 1028) for the animals treated with placebo, lurbinectedin, and gemcitabine, respectively. Tumor reductions were highly statistically significant compared with placebo (*P*<0.004). Moreover, the combination of lurbinectedin and gemcitabine produced a smaller tumor volume than either lurbinectedin (*P*=0.008) or gemcitabine (*P*=0.008) as single agents at the end of the follow-up period (day 28). The values in tumor-volume changes for each treated (*T*) and placebo (*C*) group (*T*/*C*) were reduced over the follow-up period regardless of the treatment administered (Fig. 1C). On day 28, the minimal *T*/*C* was calculated as 41.4, 42.3 and 22.7% for lurbinectedin, gemcitabine and lurbinectedin-gemcitabine, respectively. Based on the median-effect principle (Chou, 2006), the lurbinectedin-gemcitabine combination resulted in an experimental combination index (CI) value of 0.8 [at fraction affected (*F*)_a,max_=0.77], indicating a synergistic effect in SW-1990 xenografted tumors (Fig. 1E, Table 1). Fig. 1B and Fig. S1B show the tumor growth curve for animals bearing MIA PaCa-2 tumors at different dose levels. In this *in vivo* model, a marginal antitumor effect was induced by lurbinectedin or by gemcitabine, given as single agents at their respective high dose. Fig. 1D shows the *T*/*C* values for MIA PaCa-2-tumor-bearing mice treated with the highest doses of lurbinectedin, gemcitabine or the lurbinectedin-gemcitabine combination. Similar values (*T*/*C* ca. 55%) were recorded for lurbinectedin and gemcitabine as single agents during the follow-up period. However, the lurbinectedin-gemcitabine combination produced a lower *T*/*C* value than that induced by either agent alone. Thus, a net antitumor activity was seen on day 5 (*T*/*C*, 27.9%) for the combined highest doses of lurbinectedin-gemcitabine treatment (0.180+180.0 mg kg^−1^), reaching the minimal value of 23% on day 12. On the last day of the follow-up period (day 19), the lurbinectedin-gemcitabine combination still displayed a dose-dependent tumor growth inhibition, with an IQR tumor volume (mm^3^) of 759.6 (range 555.0 to 1052), 1154 (range 832.9 to 1432), 1467 (range 1041 to 1819) and 1814 (range 1365 to 2316) for the animals treated with lurbinectedin-gemcitabine at 0.180+180.0, 0.135+135.0, 0.09+90.0 and 0.045+45.0 mg kg^−1^ levels, respectively. The high-dose combination resulted in smaller tumor volume than single treatments of lurbinectedin (*P*=0.066) or gemcitabine (*P*=0.038). Analysis based on the median-effect principle (a constant CI) after treatment with the lurbinectedin-gemcitabine combination resulted in a stronger synergistic effect in mice bearing MIA PaCa-2 (CI=0.66 at *F*_a,max_=0.62) (Fig. 1F) than SW-1990 (CI=0.8 at *F*_a,max_=0.77) tumor xenografts (Fig. 1E, Table 1).

**Table 1.**
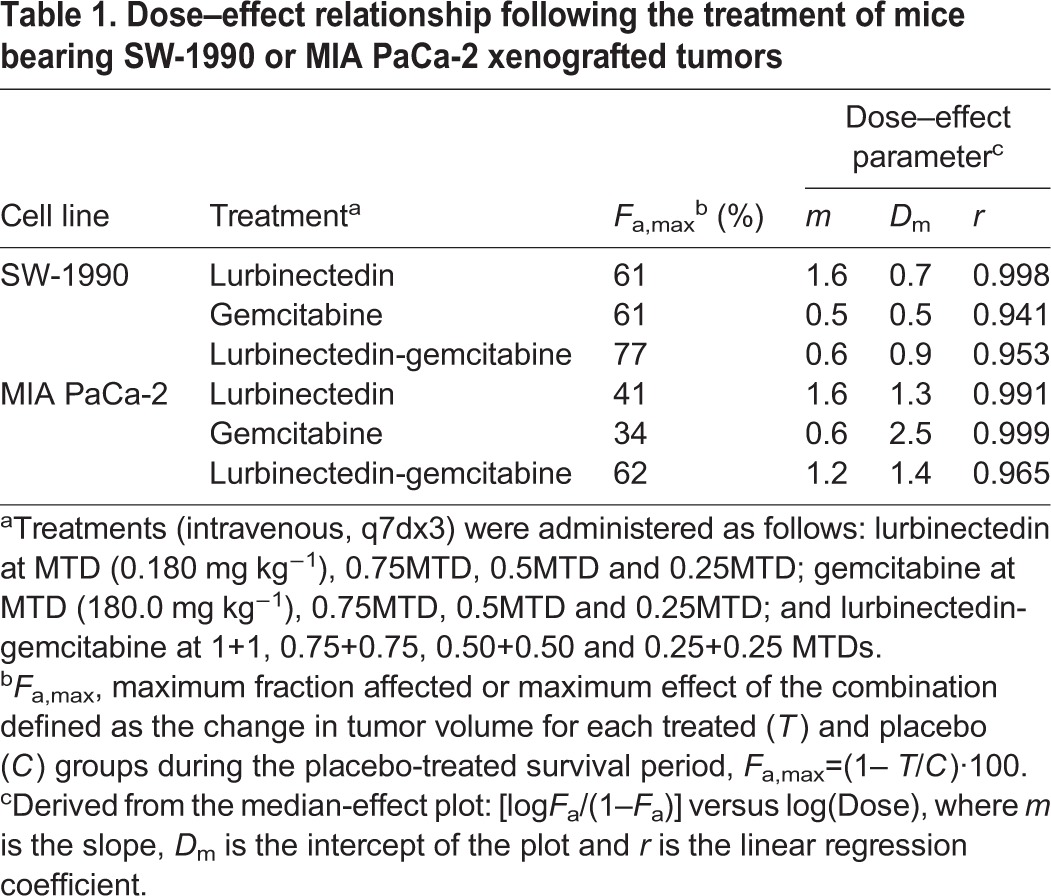
**Dose–effect relationship following the treatment of mice bearing SW-1990 or MIA PaCa-2 xenografted tumors**

### The lurbinectedin-gemcitabine synergism associates with increased DNA damage and apoptotic induction

**Fig. 2.**
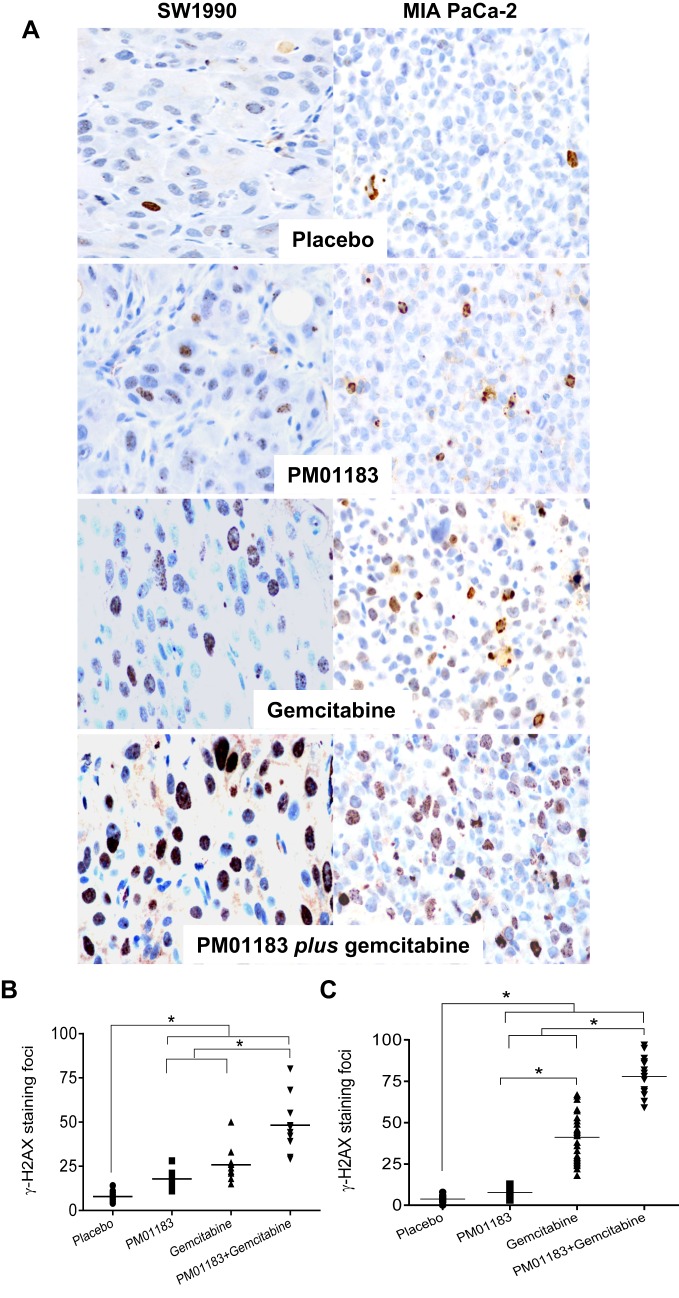
**DNA damage in tumor-bearing mice after treatment with the lurbinectedin (PM01183)-gemcitabine combination.** (A) Representative γ-H2AX-stained nuclei of SW-1990 (left panels) or MIA PaCa-2 (right panels) tumors 24 h after the administration of placebo, PM01183 (0.180 mg kg^−1^), gemcitabine (180.0 mg kg^−1^) or the combination (PM01183 plus gemcitabine, 0.180 mg kg^−1^+ 180.0 mg kg^−1^). (Original magnification, ×400.) (B,C) Quantitation of the number of γ-H2AX nuclei per µm^2^ in SW-1990 (B) or MIA PaCa-2 (C) tumors. Statistically significant differences at *P<0.01 (two-tailed Mann–Whitney U-test).

**Fig. 3.**
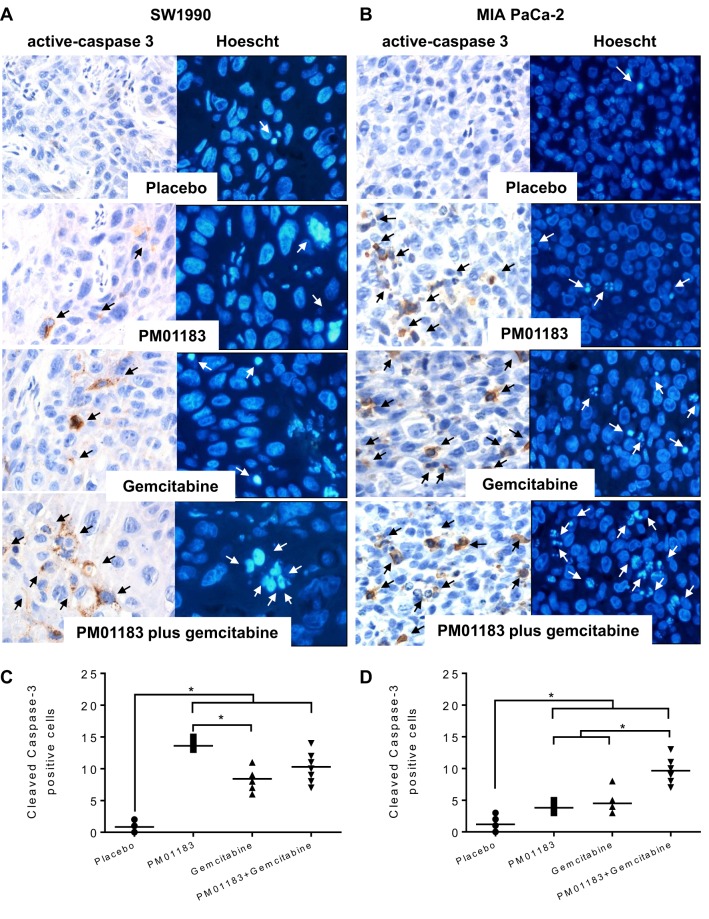
**Caspase-3 activation and apoptosis induction in tumor-bearing mice after treatment with the lurbinectedin (PM01183)-gemcitabine combination.** Representative immunohistochemistry (IHC) micrographs of cleaved (active) caspase-3 and apoptotic induction by Hoechst staining in SW-1990 (A) or MIA PaCa-2 (B) tumors, 24 h after the administration of placebo, PM01183 (0.180 mg kg^−1^), gemcitabine (180.0 mg kg^−1^) or the combination (PM01183 plus gemcitabine, 0.180 mg kg^−1^+180.0 mg kg^−1^). (Original magnification, ×400.) Arrows indicate apoptotic cells. (C,D) Quantitation of the number of cleaved-caspase-3-positive cells per µm^2^ in SW-1990 (C) or MIA PaCa-2 (D) tumors. Statistically significant differences at *P<0.01 (two-tailed Mann–Whitney U-test).

We next explored pharmacological markers that might explain the synergistic antitumor effect observed with the lurbinectedin-gemcitabine combination in SW-1990 and MIA PaCa-2 tumors. To do so, 24 h after treatment with placebo, lurbinectedin, gemcitabine or lurbinectedin-gemcitabine, tumors were dissected free and processed for γ-H2AX (a dsDNA marker), caspase-3 activation (mediator of apoptosis) and Ki67 (proliferation marker) immunohistochemistry (IHC), as well as for Hoechst 33258 nuclear staining (apoptotic figures). In SW-1990 tumors, the number (median) of γ- H2AX foci per μm^2^ was significantly increased in groups treated with lurbinectedin (19.0, *P*=0.0005), gemcitabine (23.0, *P*=0.0002) and lurbinectedin-gemcitabine (45.5, *P*=0.0002) compared with placebo (7.5). The combination also produced a higher number of γ-H2AX-stained nuclei than single treatments of either lurbinectedin (*P*=0.0002) or gemcitabine (*P*=0.0022) (Fig. 2A,B). Moreover, in these tumors, the combination treatment also increased the number of apoptotic figures compared with either agent alone. As such, 24 h after the treatment of animals bearing SW-1990 tumors, the number of condensed or defragmented apoptotic nuclei (mean±s.e.m.) per field (400× magnification) following the combination was 6.9±2.9, significantly higher than that obtained either after lurbinectedin (3.2±2.0, *P*=0.024), gemcitabine (4.1±2.1, *P*=0.024) or vehicle (3.1±0.5, *P*=0.007) treatments (Fig. 3A).

**Fig. 4.**
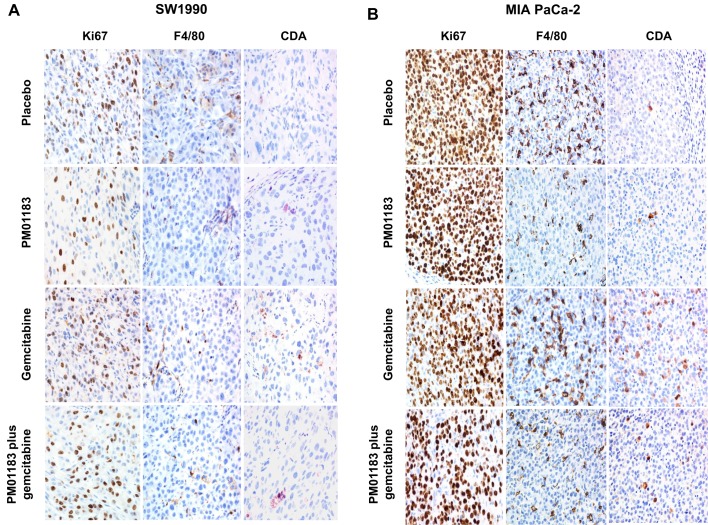
**Lurbinectedin (PM01183) induction of proliferative block or macrophage depletion and cytidine deaminase (CDA) downregulation, as a single agent or combined with gemcitabine.** Representative micrographs of proliferation rate (anti-Ki67 antibody staining), tumor associated macrophages (TAM; staining with an anti-F4/80 antibody) and CDA expression in tumor xenografts of SW-1990 (A) or MIA PaCa-2 (B) 24 h after the administration of placebo, PM01183 (0.180 mg kg^−1^), gemcitabine (180.0 mg kg^−1^) or the combination (PM01183 plus gemcitabine, 0.180 mg kg^−1^+180.0 mg kg^−1^). Gemcitabine treatment upregulates CDA. PM01183 induces TAM depletion and CDA upregulation in MIA PaCa-2 tumors, whereas it induces a proliferative block in SW-1990 tumors (original magnification 400×).

**Fig. 5.**
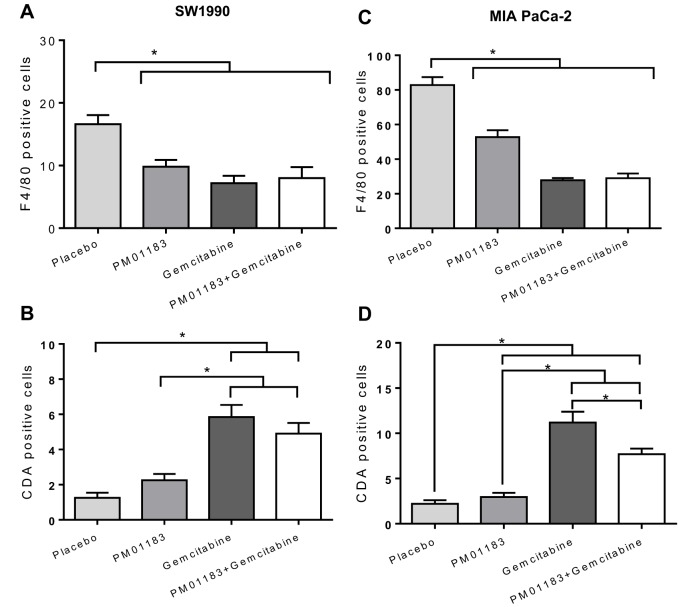
**Differences in macrophage staining and cytidine deaminase (CDA) expression among treatment groups.** Graphs depicting the number of tumor associated macrophages (TAMs; A,C) and cells with CDA expression (B,D) per µm^2^ in the complete tumor area of SW-1990 (A,B) or MIA PaCa-2 (C,D) mice 24 h after the administration of placebo, lurbinectedin (PM01183, 0.180 mg kg^−1^), gemcitabine (180.0 mg kg^−1^) or the combination (PM01183 plus gemcitabine, 0.180 mg kg^−1^+180.0 mg kg^−1^) as assessed by immunohistochemical staining. See representative images in Fig. 4. Statistically significant differences at *P<0.01 (two-tailed Mann–Whitney U-test).

In addition, in SW-1990 tumors, treatment with the lurbinectedin-gemcitabine combination increased the level of activated caspase-3 compared with either agent alone (Fig. 3A,C). Thus, 24 h after the treatment of animals bearing SW-1990 tumors, the number of cells per μm^2^ (mean±s.e.m.) showing caspase-3 activation in the combination group was 9.8±1.1, significantly higher than that obtained either after lurbinectedin (4.8±0.9; *P*=0.002), gemcitabine (3.8±0.4; *P*=0.003) or placebo (1.2±0.3; *P*=0.001) treatment (Fig. 3A,C). In addition, the treatment with lurbinectedin of mice bearing SW-1990 tumors resulted in a significant reduction in the number of proliferating cells per μm^2^ (75.0±7.7), as assessed by Ki67 expression and as compared with vehicle-treated tumors (137.0±17.8) (Fig. 4A, Fig.5A). The inhibitory effect on tumor cell proliferation in this cell line was mainly induced by lurbinectedin, because lurbinectedin-gemcitabine combination yielded to a non-significant reduction in the number of proliferative cells (87.4±4.9) as compared with the administration of lurbinectedin alone, which was, however, significantly lower than the value obtained after the treatment with gemcitabine as a single agent. (Fig. 4, Fig. 5A).

MIA PaCa-2 tumors displayed a pattern of DNA damage and apoptotic induction similar to SW-1990 tumors. Thus, in MIA PaCa-2 tumors, treatment with the lurbinectedin-gemcitabine combination group (80.0, *P*<0.0001) or gemcitabine (44.0, *P*<0.0001), and to a less extent with lurbinectedin (8.0, *P*<0.0001), induced a statistically significant increase in the number of γ-H2AX foci compared with treatment with placebo (3.5) (Fig. 2A,C). Treatment with the lurbinectedin-gemcitabine combination also resulted in a statistically significant increase in the number of γ-H2AX foci as compared with treatment with lurbinectedin (*P*<0.0001) or gemcitabine (*P*<0.0001) alone (Fig. 2A,C). Similarly, these tumors displayed a higher number of apoptotic figures (3.6±2.7) in the combination group than in the lurbinectedin-treated (1.6±0.6, *P*=0.009), gemcitabine-treated (2.3±1.1, *P*=0.034) or vehicle-treated (1.0±0.2, *P*=0.0001) group (Fig. 3B). We also observed a higher number of cells with active caspase-3 (10.3±0.7) in the combination group than in the lurbinectedin-treated (8.9±0.7, *P*<0.0001) or vehicle-treated (0.9±0.2, *P*<0.0001) group (Fig. 3B,D). In addition, whereas apoptotic induction in MIA PaCa-2 tumors treated with the combination (3.6±2.7) was, as expected, higher than the gemcitabine-treated group (2.3±1.1), the level of caspase-3 activation was higher in gemcitabine (13.6±0.4) than in the combination (10.3±0.7) group, suggesting a differential signaling pathway for apoptosis (Fig. 3B,D). Treatment with lurbinectedin or the lurbinectedin-gemcitabine combination of mice bearing MIA PaCa-2 tumors slightly reduced the Ki67 index as compared with vehicle-treated tumors, but these differences did not reach statistical significance (Fig. 4B, Fig. 5D), suggesting that, in this cell line, the synergistic effect of the combination depends mainly on the induction of cell death and not on a proliferative blockage.

### Lurbinectedin induces a stronger TAM depletion and cytidine deaminase downregulation in tumors with high levels of TAMs

The ability of lurbinectedin to selectively affect TAMs was also explored in xenografted PDA tumors. Compared with vehicle-treated animals (TAMs per μm^2^, 16.6±1.4 for SW-1990 and 82.8±4.6 for MIA PaCa-2), lurbinectedin treatment resulted in a highly significant reduction of TAMs in both SW-1990 (7.2±1.2, *P*=0.002) and MIA PaCa-2 (27.8±1.3, *P*=0.002) xenografts. The number of TAMs after lurbinectedin-gemcitabine treatment of SW-1990 (8.0±1.8) or MIA PaCa-2 (29.0±2.7) tumors was similar to that found after lurbinectedin treatment. Therefore, lurbinectedin was the main drug responsible for the depletion of TAMs in both xenograft models (Fig. 4A, Fig. 5B). Then, we evaluated the expression of cytidine deaminase (CDA) in the tumors after the administration of different treatments to mice bearing xenografts. Gemcitabine induced a significant increase in CDA-expressing cells (mean±s.e.m.) in both SW-1990 (11.2±1.2, *P*=0.0001) and MIA PaCa-2 (5.8±0.6, *P*=0.0001) tumors as compared with vehicle-treated tumors (2.2±0.4 and 1.3±0.3, respectively), whereas CDA expression in lurbinectedin-treated tumors was not different from CDA expression in vehicle-treated tumors. The treatment with the combination lurbinectedin-gemcitabine resulted in a reduced level of CDA expression in MIA PaCa-2 (7.4±0.6) and SW-1990 (4.9±0.6) tumors compared with gemcitabine-treated group (11.2±1.2, *P*=0.017; 5.8±0.6, respectively), thus suggesting that lurbinectedin might inhibit the CDA upregulation induced by gemcitabine. This effect was more evident in the high-TAM-infiltrated (MIA PaCa-2) (Fig. 4B, Fig. 5F) than in the low-TAM-infiltrated (SW-1990) tumors (Fig. 4A, Fig. 5C). Altogether, these results suggest that the depletion of TAMs and its associated CDA downregulation induced by lurbinectedin significantly contributes to the antitumor synergism of the lurbinectedin-gemcitabine combination.

### The combined treatment of lurbinectedin and gemcitabine results in a synergistic antitumor effect in patient-derived (AVATAR) PDA xenografts

**Fig. 6.**
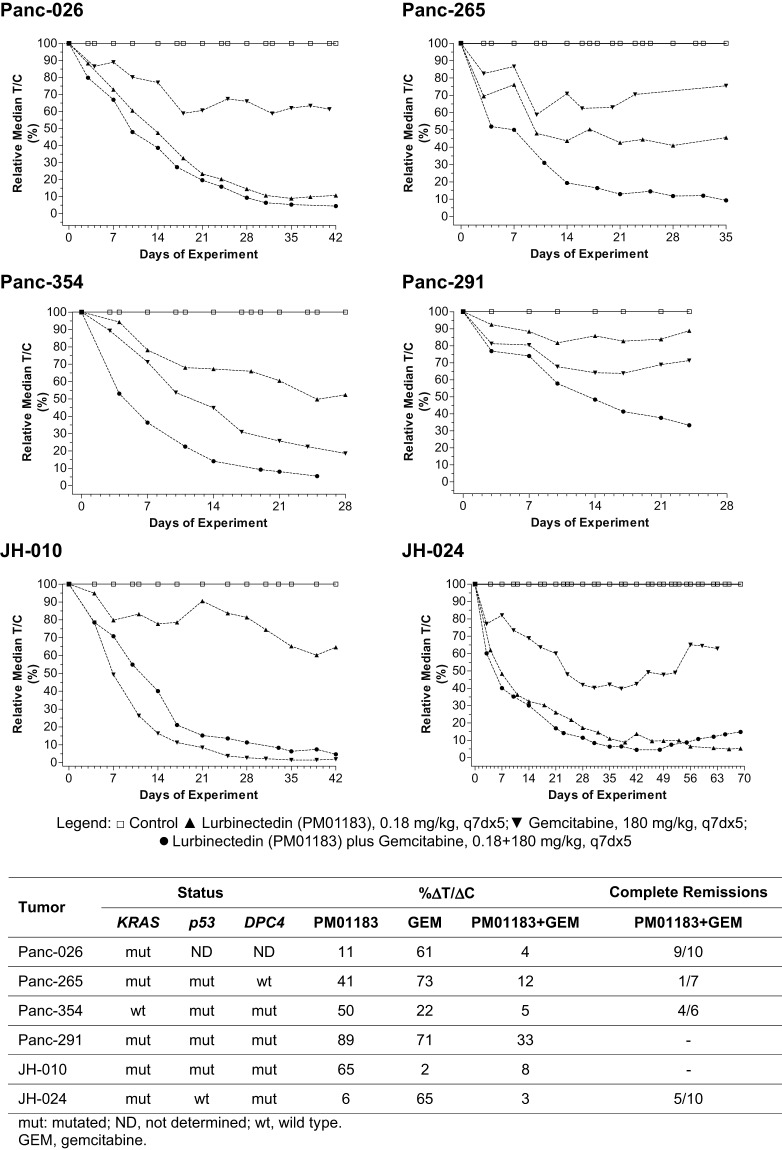
**Tumor response to treatment in athymic nude mice bearing PDA-AVATAR xenografts.** Mice bearing Panc-026, Panc-265, Panc-354, Panc-291, JH-010 or JH-024 tumors were treated with placebo, lurbinectedin (PM01183, 0.180 mg kg^−1^) or gemcitabine (180.0 mg kg^−1^) on days 0, 7, 14, 21 and 28. Treatment-induced antitumor activity was determined by the relative ΔT/ΔC (%), defined as the percentage of change in tumor size for treated (*T*) and placebo (*C*) groups in each experiment. Also, complete tumor regressions (CR) were defined when two or more consecutive tumor measurements were smaller than 63 mm^3^. DPC4, MADH4/SMAD4 gene.

Last, we evaluated the antitumor effect of the lurbinectedin-gemcitabine combination in animals bearing six different PDA-AVATAR tumors, namely Panc-026, Panc-265, Panc-291, Panc354, JH-010 and JH-024. In these experiments, intravenous treatments were administered once per week for five consecutive weeks. Fig. 6 displays the time curves of the antitumor activity (as measured by *T*/*C* values) obtained following the treatments with lurbinectedin, gemcitabine or lurbinectedin-gemcitabine for the six AVATAR models. Results showed that lurbinectedin as a single agent was active against Panc-026, JH-024 and, to a less extent, Panc-265. Gemcitabine treatment as a single agent was active against Panc-354 and JH-010, which were poor responders or insensitive to lurbinectedin treatment. Only one tumor (Panc-291) was refractory to both lurbinectedin or gemcitabine treatment. However, in four out of six tumors (Panc-026, Panc-265, Panc-291 and Panc-354), the lurbinectedin-gemcitabine combination resulted in lower *T*/*C* values than either agent alone. The lurbinectedin-gemcitabine combination induced complete tumor regression (Fig. 6, bottom) in four of the six AVATAR models. This regression lasted 19.9±8.8 (Panc-026), 14.0±0.0 (Panc-265), 22.0±18.4 (Panc-354) and 41.8±30.7 (JH-024) days.

## DISCUSSION

The aim of this study was to assess whether the combination of lurbinectedin and gemcitabine could induce a synergistic *in vivo* effect, as well as to investigate the mechanistic contribution of lurbinectedin to this synergism. As such, we found that the combination was synergistic in two PDA xenograft mouse models (derived from the human PDA cell lines SW-1990 and MIA PaCa-2) with total absence of systemic toxicity. This combination also induced complete tumor remissions in four out of six patient-derived PDA xenografts, supporting the clinical relevance of our findings. We have also explained this synergism by the contribution of two concomitant pharmacological effects, namely the induction by both drugs of DNA damage in cancer cells, and the selective depletion of TAMs by lurbinectedin in the tumor stroma.

Our results show that the combination of lurbinectedin and gemcitabine induced a significant increase of γ-H2AX levels [a surrogate marker of DSBs (Mah et al., 2011)] and clear caspase-3 activation (apoptosis induction), which were recorded in two different tumor xenografts after 24 h of drug treatment. In addition, in one of the models we also observed a marked reduction in Ki67 (a proliferation marker). These pharmacological effects could be ascribed to the activity of both drugs at the DNA level and suggest that the combination treatment synergistically enhances the antitumor effect induced by each compound. These results are not surprising because the modes of action of both drugs have been described as being the result of their interaction with DNA (Galmarini et al., 2002). As detailed previously, after binding to specific sequences on the DNA minor groove, lurbinectedin inhibits active transcription and the NER system by specific mechanisms, inducing DNA breaks and apoptosis of tumor cells. In contrast, the activity of gemcitabine was related to different mechanisms, such as inhibition of the base-excision repair (BER) system, inhibition of ribonucleotide reductase enzyme, a reduction of intracellular dCTP pool levels and incorporation into RNA (for a review, see Galmarini et al., 2002). Moreover, our findings are in agreement with previous reports showing synergism for the combination of DNA-damaging agents such as gemcitabine plus oxaliplatin, which induces radiosensitization in PDA (Morgan et al., 2008), or lurbinectedin plus cisplatin, which inhibits the growth of cisplatin-resistant ovarian tumors (Soares et al., 2011; Vidal et al., 2012). Altogether, these pharmacological events might partially explain the *in vivo* antitumor synergism induced by direct interaction between both lurbinectedin and gemcitabine with PDA tumor cells.

Our results also show that lurbinectedin reduced the amount of TAMs in PDAs. The relevance of TAMs (which can achieve up to 50% of the tumor cell mass) in tumor biology has been reported in a broad variety of different tumor types, such as PDA (Mielgo and Schmid, 2013), uterine (Kübler et al., 2014), esophageal (Sugimura et al., 2015), breast (Leek et al., 1996), ovarian (Reinartz et al., 2014), and small (Hamilton et al., 2015) or non-small (Ohri et al., 2011) cell lung cancer. Similarly, TAMs have been associated with poor prognosis and increased risk of metastasis in different series of cancer patients (Heusinkveld and van der Burg, 2011). TAMs are involved in several cancer-related processes, such as the supply of growth factors, the promotion of the formation of new vessels, and the secretion of different enzymes involved in invasion and spread of tumor cells to distant sites, as well as the induction of immunosuppression, which allows the tumor to evade the immune system (Allavena et al., 2012). The effect of lurbinectedin on TAMs was expected because, recently, Germano and Allavena demonstrated that trabectedin, a compound structurally related to lurbinectedin, induces rapid apoptosis in stromal mononuclear phagocytes, leading to the depletion of TAMs in preclinical models and sarcoma samples of patients that were treated with the drug (Allavena et al., 2013; Germano et al., 2013). This specific effect on TAMs could also explain the synergistic effect of the combination with gemcitabine in our PDA models. In this regard, Weizman et al. (2014) recently demonstrated that TAMs mediate acquired resistance of cancer cells to gemcitabine. Indeed, the authors proved that TAMs upregulate CDA, one of the enzymes that catabolize gemcitabine [2′,2′-difluoro-2′-deoxycytidine (dFdC)] in tumors into its inactive derivative, dFdU. As a consequence, the enhanced CDA activity decreases gemcitabine intracellular levels and, thus, gemcitabine-induced apoptosis. In their models, inhibition of monocyte/macrophage trafficking by a CSF1-receptor antagonist augmented the effect of gemcitabine. Our findings show an association between lurbinectedin-induced TAM depletion and CDA downregulation in PDA cells in mice treated with the combination compared with those treated with gemcitabine alone. This effect was particularly remarkable in tumors with the highest content of TAMs and was also associated with the enhanced induction of DNA damage, apoptosis and tumor shrinkage by the combination. According to these data, lurbinectedin-induced depletion of TAMs downregulates CDA expression, which is expected to lead to an increase in gemcitabine tumor levels. Therefore, we can assume that the interaction of lurbinectedin with TAMs was another contributing pharmacological factor to the lurbinectedin-gemcitabine synergism observed in the PDA models used in the present research.

Interestingly, Di Caro et al. (2016) described that increased TAM density was associated with a worse prognosis; however, gemcitabine-based chemotherapy restrained their protumor prognostic significance by reverting TAM into M1-macrophages, which activates their anticancer activity. Their and our data differ in the fact that the chemotherapy combination they used did not include a TAM-depleting agent such as lurbinectedin; therefore, the synergistic pharmacological interaction between gemcitabine and a TAM-depleting agent was not in place in their study.

In conclusion, we here elucidate the pharmacological effects that could clarify the lurbinectedin-gemcitabine synergism. This can be explained by the sum of the effects in both tumor cells and TAMs exerted by lurbinectedin and gemcitabine. Our findings also suggest that this combination could be useful for clinical indications with a strong component of macrophage infiltration such as pancreatic, breast, ovarian or lung cancer (Tang, 2013; Azria and Lemanski, 2014; Hamilton et al., 2015). Altogether, these results show that lurbinectedin can be used to develop ‘molecularly targeted’ combination strategies. With a similar rationale, future studies should address how to exploit the unique mechanistic features of lurbinectedin to combine this agent either with immunological or microenvironmental modulators or with classical chemotherapeutic agents in a more rational manner.

## MATERIALS AND METHODS

### Drugs

Lyophilized lurbinectedin (PM01183) vials (1 mg) were obtained from PharmaMar (Madrid, Spain), and gemcitabine (200 mg) vials were purchased from Lilly (Indianapolis, IN, USA).

### Cell lines and tumors

Human PDA cell lines SW-1990 (CRL-2172) and MIA PaCa-2 (CRL-1420) were obtained from ATCC (VA, USA). The cell origin is tested, authenticated and certified by the ATCC for each cell line. Cell lines were kept *in vitro* at 37°C in a humidified atmosphere of 5% CO_2_ in RPMI-1640 (SW-1190) or DMEM (MIA Paca-2) supplemented with 10% fetal bovine serum (FBS). Animal experiments were carried out in athymic mice implanted with tumor fragments previously generated in donor mice. Patient-derived PDA (PDA-AVATAR) xenografts, namely Panc-026, Panc-265, Panc-291, Panc354, JH-010 and JH-024, were obtained and perpetuated in athymic mice as previously described (Rubio-Viqueira et al., 2006).

### Animals

Female athymic Swiss *nu/nu* mice (Harlan Laboratories Inc., Italy) between 4 and 6 weeks of age were housed in individually ventilated cages on a 12-h light-dark cycle at 21-23°C and 40-60% humidity. Mice were allowed free- access to an irradiated diet and sterilized water. Design, randomization and monitoring of experiments (including body weights and tumor measurements) were performed using NewLab Software v2.25.06.00 (NewLab Oncology, Vandoeuvre-Lès Nancy, France). All animal protocols were reviewed and approved according to Generalitat de Catalonia and Sant Pau Institute Animal Care and Use Committees.

### *In vivo* evaluation of synergism of lurbinectedin and gemcitabine combination

Mice were subcutaneously implanted with fragments of SW-1990 or MIA PaCa-2 tumors. Mice bearing tumors of ca. 200 mm^3^ were then included in the *in vivo* experiment and allocated to one of 13 groups (*n*=7 per group): placebo; lurbinectedin at maximum tolerated dose (MTD; 0.180 mg kg^−1^); lurbinectedin at 0.75MTD; lurbinectedin at 0.5MTD; lurbinectedin at 0.25MTD; gemcitabine at MTD (180.0 mg kg^−1^); gemcitabine at 0.75MTD; gemcitabine at 0.5MTD; gemcitabine at 0.25MTD; lurbinectedin-gemcitabine at MTD+MTD; lurbinectedin-gemcitabine at 0.75+0.75MTD; lurbinectedin-gemcitabine at 0.50+0.50MTD; and lurbinectedin-gemcitabine at 0.25+0.25MTD. Treatments were given intravenously once per week during the placebo-treated survival time. Tumor growth was recorded two or three times per week starting from the first day of treatment (day 0), and tumor volume (*V*) was estimated according to the formula *V*=(*a*·*b*^2^)×0.5, where *a* is the length or longest diameter and *b* is the width or shortest diameter.

Treatment-induced antitumor activity was then determined by ΔT/ΔC (%), defined as a percentage of the change in tumor size for treated (*T*) and placebo (*C*) groups. The fraction affected (*F*_a_) by the treatment was calculated as 1– *T*/*C* and the combination index (CI) was determined by the CI-isobol method (Chou, 2006). At 24 h post-treatment, placebo-treated animals and highly-dosed animals (*n*=3) from lurbinectedin, gemcitabine and lurbinectedin-gemcitabine groups were sacrificed. Tumors were removed, fixed in formalin and paraffin-embedded for IHC staining.

### Assessment of caspase-3 activation, DNA damage, proliferation and macrophage depletion

Separate experiments were run for SW-1990 or MIA PaCa-2 xenografts. Mice bearing subcutaneous tumors derived from each cell line were assigned to one of four groups (*n*=3/group): placebo, lurbinectedin (0.180 mg kg^−1^), gemcitabine (180.0 mg kg^−1^) or its combination (lurbinectedin*-*gemcitabine at 0.180 mg kg^−1^+180.0 mg kg^−1^). Mice were treated with a single intravenous bolus of lurbinectedin, gemcitabine or lurbinectedin-gemcitabine. At 24 hours after drug administration, mice were euthanized, and their tumors processed for the IHC analysis to assess DNA damage, active caspase-3, TAMs or proliferation, using the antibodies and protocols described in the next section. Apoptotic induction was also assessed, following the same treatment regime by Hoechst staining of tumor sections as described below.

### Immunohistochemistry

IHC of γ-H2AX (as a DSB marker), anti-active caspase 3 (cleaved caspase-3; a mediator of apoptosis induction), Ki67 (proliferation marker), F4/80 (a mouse macrophage marker), anti-CDA (cytidine deaminase) and Hoechst 33258 (as a marker for nuclear DNA condensation associated to apoptosis) staining were determined in tumors removed at 24 h. Slide processing for IHC analysis was performed with Dako autostainer automated link 48 system (Dako Colorado Inc., CO, USA). Before IHC staining, formalin-fixed and paraffin-embedded tumor sections (4 μm) were incubated at 58°C for 60 min. They were then dewaxed in xylene, dehydrated and rinsed with phosphate-buffered saline (PBS). For γ-H2AX staining, samples were incubated at 97°C for 20 min in sodium citrate buffer (pH=6). PBS-washed samples were then blocked for endogenous peroxidase activity by treatment with hydrogen peroxide 3% for 10 min. Subsequently, samples were incubated with the primary antibody anti-H2AX (1:400, #NB100-2280, Novus Biologicals, Cambridge, UK), anti-active caspase 3 (1:300, #559565, BD Pharmigen, USA), anti-Ki67 (#GA 626, prediluted, Dako, Denmark), F4/80 (1:300, #ab6640, Abcam, Cambridge, UK) and CDA (1:300, #ab137605, Abcam, Cambridge, UK), then the mouse secondary antibody EndVision (Dako, Denmark) and subjected to immunodetection with the Envision Flex+System (Dako, Denmark) using diaminobenzidine chromogen as substrate for 5 min. Slides were counterstained with hematoxylin, ethanol-dehydrated, cleared and mounted with DPX. Hoechst 33258 (#861405, Sigma-Aldrich Co., Spain) staining was performed in Triton X-100 (0.5%) permeabilized sections. Slides were then stained with Hoechst 33258 (1:5000 in PBS) for 1 h, rinsed with water and analyzed under fluorescence microscope (λ_exc_=334 nm/ λ_em_=465 nm). The number of anti-H2AX foci or apoptotic bodies (Hoechst 33258 staining) was quantified by two independent blinded counters who recorded the number of positive nuclei per ten high-power fields (magnification 400×). We registered the number of TAMs, the number of active caspase-3 cells and the number of Ki67-positive cells per μm^2^ as well as the number of apoptotic figures per ten high-power fields (magnification 400×).

### Antitumor effect of lurbinectedin, gemcitabine and its combination in patient-derived (AVATAR) xenografts

Patient-derived pancreatic tumors (Panc-026, Panc-265, Panc-291, Panc354, JH-010 or JH-024) were subcutaneously implanted as described elsewhere (Rubio-Viqueira et al., 2006) and allowed to grow to a size of 200-300 mm^3^. Then, mice bearing tumors were randomly allocated to one of four treatment groups (*n*=6-10 group): placebo; lurbinectedin at MTD (0.180 mg kg^−1^); gemcitabine at MTD (180 mg kg^−1^); or lurbinectedin-gemcitabine. Treatments were given intravenously once per week for five consecutive weeks (7 days per 5 doses, q7dx5). Tumor volume and antitumor effect were calculated as described above. Complete tumor regressions were defined by instances in which tumor size was determined to be smaller than 63 mm^3^ for two or more consecutive measurements (Plowman et al., 1997).

### Statistical analysis

Post-treatment tumor volume data were analyzed using a non-parametric, two-tailed Mann–Whitney *U-*test. The data are presented as medians and IQR or mean±s.d. Statistical analysis and data plotting were performed using GraphPad Prism v5.02 (GraphPad Software Inc.). Synergism analysis (CI and related plots, e.g. *F*_a_-CI) was done using CompuSyn software v1.0 (ComboSyn Inc.).
